# Maternal Antibody and ASD: Clinical Data and Animal Models

**DOI:** 10.3389/fimmu.2019.01129

**Published:** 2019-05-28

**Authors:** Adriana Gata-Garcia, Betty Diamond

**Affiliations:** ^1^Center for Autoimmune, Musculoskeletal and Hematopoietic Diseases, The Feinstein Institute for Medical Research, Manhasset, NY, United States; ^2^Donald and Barbara Zucker School of Medicine at Hofstra/Northwell, Hempstead, NY, United States

**Keywords:** brain-reactive antibodies, autism spectrum disorder, neurodevelopmental disorders, sex bias, gonadal hormones, sex chromosomes, microbiome

## Abstract

Over the past several decades there has been an increasing interest in the role of environmental factors in the etiology of neuropsychiatric and neurodevelopmental disorders. Epidemiologic studies have shifted from an exclusive focus on the identification of genetic risk alleles for such disorders to recognizing and understanding the contribution of xenobiotic exposures, infections, and the maternal immune system during the prenatal and early post-natal periods. In this review we discuss the growing literature regarding the effects of maternal brain-reactive antibodies on fetal brain development and their contribution to the development of neuropsychiatric and neurodevelopmental disorders. Autoimmune diseases primarily affect women and are more prevalent in mothers of children with neurodevelopmental disorders. For example, mothers of children with Autism Spectrum Disorder (ASD) are significantly more likely to have an autoimmune disease than women of neurotypically developing children. Moreover, they are four to five times more likely to harbor brain-reactive antibodies than unselected women of childbearing age. Many of these women exhibit no apparent clinical consequence of harboring these antibodies, presumably because the antibodies never access brain tissue. Nevertheless, these maternal brain-reactive antibodies can access the fetal brain, and some may be capable of altering brain development when present during pregnancy. Several animal models have provided evidence that *in utero* exposure to maternal brain-reactive antibodies can permanently alter brain anatomy and cause persistent behavioral or cognitive phenotypes. Although this evidence supports a contribution of maternal brain-reactive antibodies to neurodevelopmental disorders, an interplay between antibodies, genetics, and other environmental factors is likely to determine the specific neurodevelopmental phenotypes and their severity. Additional modulating factors likely also include the microbiome, sex chromosomes, and gonadal hormones. These interactions may help to explain the sex-bias observed in neurodevelopmental disorders. Studies on this topic provide a unique opportunity to learn how to identify and protect at risk pregnancies while also deciphering critical pathways in neurodevelopment.

## Introduction

The increasing evidence of an immune mediated pathogenesis for neuropsychiatric and neurodevelopmental disorders has shifted the focus of epidemiologic studies to include the contribution of cytokines and brain-reactive antibodies. The brain was originally thought to be an immune privileged organ due to the presence of the blood brain barrier (BBB), a structure composed of endothelial cells knit together by tight junctions and supported by astrocytic endfeet (1). We now know that even though the BBB isolates the central nervous system (CNS) from factors in the blood, it is a dynamic semipermeable structure. Immune molecules including antibodies can access the CNS during both physiologic and pathologic states. Even though antibodies cannot cross the BBB and access brain tissue in healthy adults, these molecules may cross the BBB during *in utero* development when the BBB is immature and more permeable (2). Alternatively, antibodies can penetrate the adult brain when there is a BBB breach as occurs during inflammation (3–6) or at sites of limited BBB protection such as the choroid plexus. Factors affecting BBB integrity include: trauma, ischemia, stress, aging, antibodies, and specific agonists of endothelial cell receptors, such as cytokines, complement, and antibodies themselves (7–12). Once in the CNS, antibodies can lead to pathology if they recognize antigens expressed in the brain or spinal cord. In this review we discuss how maternal brain-reactive antibodies affect fetal brain development, contributing to the risk of neuropsychiatric and neurodevelopmental disorders. We focus on antibodies implicated in Autism Spectrum Disorder (ASD) and propose a role for the microbiome, sex chromosomes and gonadal hormones in determining the susceptibility to the effects of maternal antibody and the development of neurodevelopmental disorders.

## Brain-reactive antibodies

Antibodies that recognize CNS antigens are primarily detected in three settings: autoimmune disease (AD), paraneoplastic syndromes, and infectious diseases (13). Individuals with AD in which B cell tolerance is impaired can harbor brain-reactive antibodies with the development of neurological and neuropsychiatric disorders as seen in Systemic Lupus Erythematosus (SLE) (5, 14–20), celiac disease (21, 22), and Neuromyelitis Optica (NMO) (23–26). Due to the fact that the BBB sequesters brain antigen from the immune system, these brain-reactive antibodies may be produced against non-CNS antigens, but cross-react with structurally similar epitopes in the CNS. In paraneoplastic syndromes brain cross-reactive antibodies can result from an immune response to tumor antigens that are routinely expressed by brain cells but only by non-brain cells under pathologic states. These antibodies can trigger neurologic symptoms (27), a phenomenon that has been described in breast cancer (28, 29), testicular tumors (28), small-cell lung cancer (28, 30), ovarian teratoma (31, 32), and more (33–36). Finally, exposure to microbial antigens can stimulate the production of antibodies that cross-react with CNS antigens, a process known as molecular mimicry. Infection with HTLV-1 (37, 38), Trypanosoma brucei (39–41), and group A β-hemolytic streptococcus (42–47) has been shown to produce antibodies that cross-react with brain antigens and cause neurologic disorders.

The potential for pathology to arise from brain-reactive antibodies accessing the brain parenchyma depends on multiple factors. Vulnerability to the brain-reactive antibodies requires that the anti-brain antibody be present in the CNS at a time when the antigen is expressed. Furthermore, when a BBB breach is necessary for the antibody to penetrate the brain parenchyma, the nature of the BBB insult will restrict access to specific regions of the brain, determining whether the antibody will encounter its cognate antigen. For instance, lipopolysaccharide (LPS) causes a BBB breach in the hippocampus in mice (5) while epinephrine results in a BBB breach in the amygdala (4). Additionally, genetic variants and differences in protein expression between males and females (48–50) may also influence brain vulnerability to immune assault.

The symptoms and deficits observed in disorders caused by brain-reactive antibodies are dependent on the antigen recognized and its distribution in the brain. For example, antibodies to ribosomal P protein (anti-P antibodies) present in SLE patients cross-react with a brain antigen termed neuronal surface P antigen (NSPA) (19). While these antibodies bind to several regions of the mouse brain, when injected into the lateral cerebral ventricles they lead to smell alterations (51) and depression-like behavior (52), implicating the piriform cortex, the cingulate cortex, and the hippocampus. Furthermore, upon LPS-induced BBB breach, which enables access to the hippocampus, these antibodies cause memory impairment in mice (15). SLE anti-DNA antibodies that cross-react with N-methyl-D-aspartate receptors (NMDAR) termed DNRAb cause cognitive impairment (5) and an abnormal stress response (4) in mice, consistent with the function of NMDARs in the hippocampus and the amygdala, respectively. While NMDARs are expressed in both the hippocampus and the amygdala, the regional selectivity of symptoms observed in the mouse models is determined by the agent used to compromise BBB integrity; cognitive impairment is seen only when LPS damages BBB integrity in the hippocampus while the abnormal stress response is seen with exposure to epinephrine and antibody penetration of the amygdala. This example highlights the importance of antigen distribution and the region of BBB breach.

## Maternal brain-reactive antibodies and fetal brain development

Brain-reactive antibodies have the potential to alter brain development *in utero* (53), resulting in damage that can be persistent (54) and lead to neurodevelopmental and neuropsychiatric disorders in the offspring. It has been proposed that AD-related impaired B cell tolerance in women can lead to the production of these antibodies. Indeed, ADs are more prevalent in mothers of children with neurodevelopmental and neuropsychiatric disorders including ASD (Table 1). A study by our group showed that mothers of children with ASD who had brain-reactive antibodies were significantly more likely to harbor anti-nuclear antibodies (53%) than those lacking brain-reactive antibodies (anti-nuclear antibody = 13.4%) and unselected women of child-bearing age (anti-nuclear antibody = 15%), suggesting a maternal predisposition to producing auto-antibodies. We also observed a significantly greater prevalence of AD in mothers of children with ASD who were also positive for brain-reactive antibodies [rheumatoid arthritis (RA) = 3.86%; SLE = 2.22%] compared to those lacking brain-reactive antibodies (RA = 1.45%; SLE = 0.16%) (67).

**Table 1:** Family history of autoimmune diseases and increased risk of neurodevelopmental/neuropsychiatric disorders.

	**Associations with autoimmune disease (AD)**	**Prevalence/incidence of Neurodevelopmental/neuropsychiatric disorders (ND)**		**Family history of AD**		**ND sex bias linked to AD**	**References**
		**AD offspring**	**Control offspring**	**ND**	**NT**		
Developmental problems	Maternal SLE	Hyperactivity = 13.1% Attention problems = 15.7% Reading difficulties = 21.6%	Hyperactivity = 1.3% Attention problems = 6% Reading difficulties = 9.3%			Male bias	(55)
Learning Disabilities (LD)	Increased risk of LD and maternal anti-Ro/La Abs [OR = 5.74 (95% CI, 1.39–23.74)] and SLE disease flares [OR = 9.43 (95% CI, 1.32–67.24)] during pregnancy	26%	7%			Male bias	(56)
	Maternal SLE and increased risk of impairments in learning and memory [OR = 3.45, 95% CI of OR (1.25, 9.09), *P* = 0.02]	54.9%	30.4%				(57)
Tourette Syndrome (TS)	Maternal AD and increased incidence of TS [IRR = 1.22 (95% CI, 1.01–1.48)]	2.25 per 10,000 person years	1.86 per 10,000 person years			Male bias	(58)
PDD	1st degree relative with a history of AD Family history of Hashimoto's thyroiditis and rheumatic fever			30.7%	11.9%		(59)
ADHD	Elevated maternal TPOAbs during pregnancy and increased risk of ADHD [OR = 1.77 (95% CI, 1.15–2.72)						(60)
ASD	Maternal RA and increased incidence of ASD [IRR = 1.70 (95% CI, 1.07–2.54)] Maternal celiac disease and increased incidence of ASD [IRR = 2.97 (95% CI, 1.27–5.75] Family history of type 1 diabetes and increased incidence of ASD [IRR = 1.78 (95% CI, 1.16–2.61)]						(61)
	AD in 1st degree relative and increased risk of ASD [OR 6.0] Maternal AD and increased risk of ASD [OR = 8.8]			1st degree relative AD = 21% Maternal AD = 16% RA = 46%	1st degree relative AD = 4% Maternal AD = 2% RA = 26%		(62)
	Maternal psoriasis and increased risk of ASD [OR = 2.7 (95% confidence interval, 1.3–5.8)]			Psoriasis = 2.7%	Psoriasis = 1.0%		(63)
	Family history of AD and increased risk of ASD [OR = 6, 95% CI, 2.5–14.1)]			AD = 40%	AD = 10%		(64)
				Family history AD = 45%	Family history AD = 10%		(65)
	Maternal SLE and increased risk of ASD [OR = 2.19 (95% CI 1.09–4.39)]	1.4%	0.6%				(66)

*AD, autoimmune disease; ND, Neurodevelopmental/neuropsychiatric disorders; NT, Neurotypical development; SLE, Systemic lupus erythematosus; LD, learning disabilities; Abs, antibodies; TS, Tourette Syndrome; PDD, Pervasive Developmental Disorders; ADHD, Attention deficit/hyperactivity disorder; TPOAbs, thyroid peroxidase antibodies; ASD, Autism Spectrum Disorder; RA, Rheumatoid arthritis*.

The BBB is immature during fetal development, presenting a uniquely vulnerable temporal window when antibodies can enter the CNS (2) while critical neurodevelopmental events are taking place. The antibodies that access the fetal brain are produced by the mother and are transported by the neonatal Fc receptor (FcRn) across the placenta into fetal circulation starting on week 13 of human gestation (68–70). Once maternal brain-reactive antibodies access the fetal circulation they may result in pathology if the timing of antigen expression also coincides with the period when the fetal BBB is permeable to IgG. Presence of maternal brain-reactive antibodies during pregnancy alone is, therefore, not enough to result in pathology, perhaps contributing to the fact that some mothers of neurotypically developing (NT) children also have anti-brain antibodies.

When assessing the potential for maternal brain-reactive antibodies to cause neurodevelopmental disorders we must also be mindful that the women harboring these antibodies will not necessarily present with neurological deficits resulting from antibody exposure as their BBB is likely to be intact or the impact of the antibody may be developmentally determined. Moreover, the deficits caused by *in utero* antibody exposure may be transient or long-lasting, and consequently, not all children born to mothers with brain-reactive antibodies will present post-natally with detectable symptoms. Transient insults may be compensated for by plasticity mechanisms in the brain during gestation or post-natally. Furthermore, it is possible for deficits to be present only while the pathogenic antibody has access to the brain parenchyma, which is limited after birth by the maturation of the BBB (2) and the disappearance of maternal antibodies in the circulation of newborn infants (71). Lastly, symptomatology from the neurodevelopmental effects of maternal brain-reactive antibodies may appear later in life or may become apparent only if other stressors are present (72).

## Maternal brain-reactive antibodies and ASD

ASD are a group of neurodevelopmental conditions characterized by impaired communication and social interactions, repetitive behaviors, and restricted interests or activities (DSM-V) (73). They are four times more likely to be diagnosed in males compared to females. Both prevalence and incidence of ASD are increasing, with a current estimate of 1 in 59 children being affected (74). The etiology of ASD is not completely understood; hundreds of genes have been associated with ASD (75, 76) but these account for just 10–20% of the diagnosed cases (77). Furthermore, twin studies indicate that only 37% of the susceptibility to ASD is due to genetic heritability (78). These data suggest that environmental factors also play an important role in determining the susceptibility to ASD. Maternal brain-reactive antibodies present *in utero* represent a potential environmental risk factor for ASD. Several groups have identified brain-reactive antibodies in mothers of children with ASD which are either absent or found at lower frequency in mothers of unaffected children (Table 2). Dalton et al. (79) showed that, when injected into pregnant mice, brain-reactive serum from a mother of a child with autism and a child with severe specific language disorder led to decreased exploration, deficient motor coordination, and altered cerebellar metabolites in the offspring compared to the offspring of mice given sera from mothers of NT children. In a study in which blood samples were collected mid-pregnancy, Croen et al. (80) suggested a direct pathogenic role for the antibodies. A significantly higher prevalence of reactivity to proteins from brain lysates of 39 and 73 kDa was detected in mothers of children with ASD compared to the general population control group. Moreover, this pattern of reactivity was seen in mothers of children with early onset ASD.

**Table 2:** Maternal brain-reactive antibodies linked to ASD.

**Target**	**Reactivity**	**Phenotypic associations in humans**	**References**
Cerebellar Purkinje cells and brainstem neurons	Adult rat and P1 mouse brains by IHC Binding to NB-1 cells (cells derived from a human neuroblastoma)	Serum obtained from a mother of a child with ASD and a child with a language disorder	(79)
LDH-A, LDH-B	37 kDa band on WB using human and rhesus macaque fetal brain proteins	ASD with behavioral regression Within individuals with ASD, 1. Abnormal sleep/wake cycle 2. Deficits in verbal and non-verbal language acquisition 3. Increased stereotypical behaviors on the ABC	(81–86)
STIP1 (target for upper band CRMP1, CRMP2 (target for lower band)	73 kDa band on WB using human and rhesus macaque fetal brain proteins	Within individuals with ASD, 1. Verbal language deficits 2. Delayed onset of social smile	(82–86)
LDH reactivity in combination with STIP1 or STIP1/CRMP1	37/73 kDa band combination on WB using human and rhesus macaque fetal brain proteins	1. Pattern only observed in mothers of children with ASD 2. ASD with behavioral regression 3. Greater total cerebral volume Within individuals with ASD, 4. Lower score for expressive language measured by MSEL 5. Increased stereotypical behaviors on the ABC	(81–83, 85–87)
YBX1	39 kDa band on WB using human and rhesus macaque fetal brain proteins	Early onset ASD	(80, 82–86)
YBX1 reactivity in combination with STIP1 or STIP1/CRMP1	39 kDa/73 kDa band combination on WB using human and rhesus macaque fetal brain proteins	1. Early onset ASD 2. Decreased motor skills scores measured with VABS 3. Increased irritability on the ABC compared to children with ASD born to mothers without these reactivities	(80, 82, 83, 85, 86)
Cypin or GDA	44 kDa band on WB using rhesus macaque fetal brain proteins		(83, 84)
Not identified	36 kDa/39 kDa band combination on WB with human fetal and rat embryonic brain proteins	ASD with developmental regression.	(88)
Not identified	Low molecular weight bands (~20–25 kDa) High molecular weight band (larger than 250 kDa) on WB using fetal rat brain proteins		(89)
Yo	Immunoblot with recombinant protein		(90)
Amphiphysin	Immunoblot with recombinant protein		(90)
Caspr2	Isolation and cloning of single human memory antigen-specific B cells Live cell-based assay using HEK-293 cells expressing tGFP-Caspr2 Binding to adult wild type but not to adult CNTNAP2 KO mouse brain by IHC		(91)

*P, ponstnatal day; IHC, Immunohistochemistry; NB, neuroblastoma; ASD, Autism Spectrum Disorder; WB, Western blot; LDH-A, lactate dehydrogenase A; LDH-B, lactate dehydrogenase B; STIP1, stress-induced phosphoprotein 1; CRMP1, CRMP2, collapsing response mediator protein 1 and 2; MSEL, Mullen Scales of Early Learning; NT, Neurotypical development; YBX1, Y-box bonding protein 1; VABS, Vineland Adaptive Behavioral Scales; ABC, Aberrant Behavioral Checklist; GDA, Guanine deaminase; HEK, Human Embryonic Kidney cells; tGFP, Turbo green fluorescent protein; Caspr2, Contactin-associated protein-like 2; CNTNAP2, Contactin-associated protein-like 2 gene; KO, Knockout*.

Some of these maternal brain-reactive antibodies have been found to have antigenic specificity for proteins with potential neurodevelopmental roles including: lactate dehydrogenase A and B (LDH-A, LDH-B) (37 kDa band), Y-box bonding protein 1 (YBX1) (39 kDa band), stress-induced phosphoprotein 1 (STIP1) (upper 73 kDa band), collapsing response mediator protein 1 and 2 (CRMP1, CRMP2) (lower 70 kDa band), and guanine deaminase (GDA) (44 kDa band) (83, 84). Maternal reactivity to LDH alone or in combination with reactivity to CRMP1/CRMP2/STIP1 has been associated with a greater risk of ASD. Reactivity to LDH, STIP1, and CRMP1 together (the 37 and 73 kDa combined bands) was the most specific pattern for ASD as it was detected in mothers of children with ASD but not in mothers of NT children (81, 83). Furthermore, presence of this antibody combination was associated with an increased risk of behavioral regression in ASD (81) and impairments in expressive language (82).

Animal studies have shown that exposure to maternal brain-reactive antibodies *in utero* can permanently alter the brain during development and cause sustained behavioral and cognitive deficits akin to those observed in ASD (Table 3). Martínez-Cerdeño et al. (96) and Ariza et al. (97) used a single intraventricular embryonic injection model to assess the effects of brain-reactive antibodies recognizing LDH/STIP1/CRMP1 (antigens of molecular weights 37 and 73 kDa) on fetal brain development. These antibodies stimulated the proliferation of stem cells in the subventricular zone (SVZ) of the neocortex and ganglionic eminence, increased adult brain size and weight, and enlarged adult cortical neuron volume (96). Additionally, anti-LDH/STIP1/CRMP1 antibodies decreased basal dendritic arborization in layer V neurons of the frontal cortex and reduced the dendritic spine number and density in several brain regions (97). Complementing these studies, mice exposed *in utero* to maternal brain-reactive antibodies recognizing LDH/STIP1/CRMP1 showed ASD-like characteristics including increased anxiety-like behaviors (93), impaired social interactions (95), longer bouts of spontaneous grooming (95), increased digging (95), and delayed motor and sensory development (93).

**Table 3:** Animal studies demonstrating that *in utero* exposure to maternal brain-reactive antibodies can permanently alter the brain, leading to behavioral and cognitive deficits.

**Target**	**Animal model**	**Characteristics**	**References**
Cerebellar Purkinje cells and brainstem neurons	Passive transfer: daily maternal serum injections into pregnant mice from E10 to E17	Decreased exploration Altered motor coordination Cerebellar metabolite abnormalities.	(79)
Unknown	Passive transfer: IV injections of pooled maternal IgG to pregnant rhesus macaque on gestation days 27, 41 and 55	Hyperactivity Increased stereotypies	(92)
LDH/STIP1/CRMP1 (antigens of molecular weights 37 and 73 kDa)	Passive transfer: single IV injection of purified maternal IgG into pregnant mice on E12	Delayed pre-weaning motor and sensory development. Increased number of USVs on P8 Males had a longer total USV duration on P8 Increased anxiety-like behaviors in males Slightly shorter social interaction in males	(93)
	Passive transfer: IV maternal IgG injection into rhesus macaque throughout pregnancy	Aberrant social behaviors Enlarged brain volume due to increases in white matter in male offspring.	(94)
	Single intraventricular maternal IgG injection into E14 mouse embryos	Increased repetitive behaviors measured as digging in the marble test and grooming Impaired social interactions	(95)
	Single intraventricular maternal IgG injection into E14 or E16 mouse embryos	Greater number of proliferating stem cells in the SVZ of the neocortex and ganglionic eminence Increased adult brain size and weight Increased adult cortical neuron some volume	(96)
	Single intraventricular maternal IgG injection into E14 mouse embryos	Decreased basal dendritic arborization in layer V pyramidal neurons of the frontal cortex Reduced the dendritic spine number and density in several brain regions	(97)
	Endogenous production: female mice were immunized prior to pregnancy with antigenic peptides recognized by anti- LDH/STIP1/CRMP1 antibodies.	Impaired social interactions Impaired social communication measured by USVs neonatally and as adults. Increased repetitive behaviors measured as grooming	(98)
Unknown	Passive transfer: daily IP injections of pooled maternal IgG to pregnant mice from E13 to E18.	Hyperactivity Increased anxiety Impaired social interactions Increased IL-12 levels on E16 and microglia activation on E18 fetal brains.	(99)
	Passive transfer: daily IP injections of pooled maternal IgG to pregnant mice from E13 to E18.	Greater cell proliferation in the SVZ and SGZ post-natally. Decreased cortical cell survival post-natally.	(100)
Caspr2	Passive transfer: single IV injection of anti-Caspr2 IgG to pregnant mice on E13.5	Male fetuses: 1. Thinner cortical plate 2. Fewer proliferating cells in the VZ 3. Reduced number of neurons in the entorhinal cortex Adult males: 1. Decreased number of GABAergic neurons in the hippocampus 2. Decreased dendritic arborization and spine density in CA1 pyramidal neurons 3. Increased stereotypic behaviors: increased digging measured as digging in the marble test 4. Impaired flexible learning 5. Impaired social interactions	(91)
DNA and NMDAR	Endogenous production: female mice were immunized prior to pregnancy with a peptide mimetope of DNA. Passive transfer: single IV injection of NMDAR reactive IgG on E14 to pregnant mice	Fetuses: 1. Increased cortical cell death and proliferation 2. Thinner cortical plate • Adults: 1. Decreased cortical neuron size 2. Decreased cortical volume 3. Cognitive impairments in males	(101)
	Endogenous production: female mice were immunized prior to pregnancy with a peptide mimetope of DNA.	Increased female fetal death rate	(102)

*E, Embryonic day; IV, intravenous; LDH, lactate dehydrogenase; STIP1, stress-induced phosphoprotein 1; CRMP, collapsing response mediator protein 1; SVZ, subventricular zone; USVs, ultrasonic vocalizations; P, ponstnatal day; IP, intraperitoneal; SGZ, subgranular zone; Caspr2, Contactin-associated protein-like 2; VZ, ventricular zone; NMDAR, N-Methyl-D-aspartate receptor*.

A limitation of these animal studies is the timing of exposure to the maternal brain-reactive antibodies. Maternal brain-reactive antibodies associated with ASD in humans are likely to be present throughout the pregnancy. These studies did not simulate these conditions; they used a single injection into the pregnant mice at mid-gestation or a single intraventricular injection into the embryos. To address this, Jones et al. (98) completed a study in which female mice were immunized prior to pregnancy with antigenic peptides recognized by anti- LDH/STIP1/CRMP1 antibodies. The offspring mice exposed to endogenous maternal anti-LDH/STIP1/CRMP1 antibodies displayed fewer social interactions as juveniles and adults, increased repetitive behaviors/stereotypies assessed through the number and length of grooming bouts, and impaired social communication measured by ultrasonic vocalizations (USVs) neonatally and as adults.

In other studies identifying pathologic maternal brain-reactive antibodies, Singer et al. (88) found that, compared to mothers of NT children, mothers of children with ASD have a significantly higher prevalence of antibodies reactive to a 36 kDa protein present in rat embryonic and human fetal brain. They also noted a trend for a higher prevalence of antibodies recognizing human fetal proteins at 39 kDa in mothers of children with ASD compared to mothers of NT children. Presence of either of these reactivities was significantly associated with behavioral regression in children with ASD. Intraperitoneal administration of the purified maternal ASD-IgG to pregnant mice led to hyperactivity, increased anxiety, and shorter social interactions in the adult offspring relative to the offspring of pregnant mice given IgG from mothers of NT children or saline (99). Preliminary fetal brain studies suggested a role for microglia and IL-12 in the pathological mechanism of the ASD-IgG induced behavioral irregularities (99). Further assessment of the pathological mechanism of these antibodies showed greater cell proliferation in the subventricular and subgranular zones and decreased post-natal day (P)1-born cell density, suggesting reduced survival, in layers 2–4 of the frontal and parietal cortex (100). Due to the similarity in antigen size and the association with behavioral regression, it is possible that the samples from the Braunschweig and Singer studies contain antibodies that recognize the same proteins at 36–39 and 73 kDa, and that these antibodies represent contributors to ASD risk in the general population. Determining the antigenicity of the antibodies identified by Singer et al. (88) will be necessary to resolve this question.

Animal studies of the pathogenic role of ASD-IgG have generally used IgG that was pooled from several mothers of children with ASD or endogenous polyclonal antibody following an immunization protocol. Thus, it has not been possible to identify the antibodies that are pathogenic from those that are not. Furthermore, specific ASD-like characteristics in the animal studies may result from exposure to distinct monoclonal antibodies. There is also likely to be a different proportion of potentially pathogenic antibodies in the pooled sample compared to the composition present in each of the mothers. As a result, the effects of antibodies at low concentration might be obscured by those at high concentration or they may not be detectable because the threshold concentration of antibody necessary to produce pathology may not have been reached. Moreover, the studies discussed above do not definitively identify the targeted antigen as it remains possible that the critical antibodies bind not only to the identified antigens (most of which are intracellular) but also cross-react with a neuronal membrane antigen. We addressed this concern by developing a protocol to generate monoclonal brain-reactive antibodies from mothers of children with ASD and a brain-reactive serology.

One of the monoclonal antibodies that we generated recognizes the extracellular domain of Caspr2, a protein encoded by the gene Contactin Associated Protein-Like 2 (CNTNAP2). Caspr2 is a cell-adhesion molecule expressed in the spines, dendrites, axons, and soma of neurons (103, 104). Both rare and common variants of CNTNAP2 have been linked to an increased risk of ASD or ASD-related endophenotypes including language delay and developmental language disorders (105–115). Furthermore, CNTNAP2 deficient mice exhibit ASD-like phenotypes including increased repetitive behaviors, and impaired communication and social interactions (116). The CNTNAP2 deficient mice also suffer from seizures, show neuronal migration abnormalities and have ectopic neurons in the corpus callosum (116), similar to cortical dysplasia-focal epilepsy (CDFE) syndrome, a syndromic form of ASD associated with mutant CNTNAP2 (114).

Given the link between mutations in CNTNAP2 and ASD in human pedigrees and the presence of ASD-like phenotypes in CNTNAP2 deficient mice, we asked whether exposure to monoclonal anti-Caspr2 antibody (C6) *in utero* leads to ASD-like characteristics in mice (91). Indeed, *in utero* C6 exposure led to a thinner cortical plate and fewer proliferating cells in the ventricular zone, and to a reduction in the number of neurons in the entorhinal cortex and in the number of GABAergic neurons in the hippocampus of adults. We also observed decreased dendritic arborization and a reduced spine density in CA1 pyramidal neurons in adult mice exposed to C6 *in utero* when compared to the controls. Finally, these mice showed ASD-like behavioral abnormalities such as stereotypic behaviors, impaired flexible learning, and impaired social interactions. Interestingly, effects of C6 were only detected in male mice.

## Maternal brain-reactive antibodies and sex-bias

Neuropsychiatric conditions often display a bias for one sex over the other. Neuropsychiatric conditions diagnosed earlier in life are more frequently diagnosed in males while those manifesting during puberty or later in life show a female preponderance. For example, there is a male bias in ASD, ADHD, dyslexia, Tourette Syndrome, and learning disabilities [reviewed by (117)]. Conversely, anorexia nervosa and internalizing disorders such as depression (118) and anxiety are more prevalent in females (119). Neuropsychiatric conditions that are diagnosed earlier in life have been proposed to have a neurodevelopmental origin (119). Given the unique conditions during pregnancy that allow for the *in utero* environment including maternal antibodies and cytokine levels to influence development, it is reasonable that early-onset neuropsychiatric disorders are more frequent in children of mothers with AD (Table 1).

A sex-bias has indeed been described in multiple studies of the effects of maternal brain-reactive antibodies on development and behavior. For example, we observed a significant male bias for all the fetal brain developmental and adult behavioral effects of the C6 anti-Caspr2 antibody (91). Females exposed to C6 *in utero* were not affected while males developed disrupted brain anatomy and ASD-relevant behaviors. The animal studies of maternal anti-LDH/STIP1/CRMP antibodies have also noted a male-bias in ASD-like traits including impaired communication and social interactions (93, 98). Lastly, Wang et al. (102) observed a significantly higher rate of fetal death after embryonic day (E) 15 in female offspring exposed to SLE DNRAb compared to males. While females had a greater fetal death risk, males in this model were born with cognitive impairment (101). This difference between the sexes suggests that sex-dependent factors play a role in determining not only the impairments resulting from maternal brain-reactive antibody exposure but also the severity of the outcomes.

Sex chromosomes and gonadal hormones may influence the susceptibility to maternal brain-reactive antibodies. Sex chromosome genes contribute to sexual dimorphisms, including sex-specific patterns of brain development and function, independently from gonadal hormone influences (50, 120). For example, sex chromosomes have been implicated in the density of vasopressin fibers (121), the number of tyrosine hydroxylase expressing neurons (120, 122), social interactions (123, 124), aggression (125–127), and anxiety (128). Genes found in “sex specific” regions in the X and the Y chromosomes may account for some of these sexual dimorphisms. These genes are highly expressed in the brain (48, 50) and show expression, spatial, and temporal differences between sexes (50). Furthermore, sex differences may be in part explained by gene dosage as some genes on the X chromosome escape inactivation (129–131), and X chromosome imprinting can affect gene expression in the brain (132, 133). Indeed, X chromosome imprinting has been associated with social impairment in Turner syndrome (134). Higher expression of these sex chromosome genes may be protective if they encode the antigens recognized by the maternal brain-reactive antibodies when binding of the antibody leads to protein internalization and partial loss of function but is only pathogenic if protein expression falls below a certain threshold. Conversely, if antibody interaction with its cognate antigen induces cell signaling cascade activation or apoptosis, then the sex with higher expression would be at an increased risk for developing the antibody-induced phenotypes. Furthermore, temporal differences in gene expression between sexes may be important as antibody exposure must coincide with this period in order to cause pathology. Moreover, the effects of sex chromosome genes on the susceptibility to maternal brain-reactive antibodies may be indirect if the proteins encoded modulate the expression or activity of the proteins recognized by the antibodies. For instance, the DNRAb mediated female fetal loss may be in part due to sex chromosome genes acting as regulating factors and contributing to the higher expression of the NMDAR subunit NR2A in the female brainstem by E17 (102).

Sex hormones also influence brain development, aiding normal maturation of the fetal brain or altering normal development. Estrogen has neurotrophic and neuroprotective functions including modulation of neuronal apoptosis, migration, and spinogenesis and neurite growth (135–140). Estrogen administration during fetal development leads to masculinization of mouse neural pathways and behaviors (141, 142). Human fetal testosterone has been linked to narrow interests (143) and greater impairments in social skills and empathy in offspring (143–145). Additionally, treatment with estrogen reversed or mitigated some of the ASD-relevant behavioral phenotypes in animal models of ASD, importantly, CNTNAP2 mutant zebrafish (146) and Reeler heterozygous mice (147). As estrogen treatment showed a decrease in phenotypic behavior, these data suggest that estrogen may have a protective role in ASD and could therefore account for the lower prevalence in females. Overall, gonadal hormones modulate processes in brain development and maturation that could lead to compensation for or exacerbation of the pathologic effects of maternal brain-reactive antibodies. The specific effects of individual gonadal hormones are likely to depend on the neural pathways affected by the antibodies.

Given the sex-bias of neurodevelopmental and neuropsychiatric disorders in humans, understanding what causes maternal brain-reactive antibodies to affect preferentially one sex over the other will aid in our understanding of the pathological mechanisms of these conditions while also providing information that could lead to the discovery of new treatments. Of note, exposure to gonadal hormones *in utero* in litter-bearing animals differs from that in humans due to the “intrauterine position phenomenon.” Fetuses between two males will be exposed to higher levels of testosterone while fetuses between two females will be exposed to higher levels of estrogen [reviewed by (148)]. Consequently, post-natal sexually dimorphic characteristics including brain anatomy and behavior are influenced by fetal position [reviewed by (148)]. Nonetheless, the importance of gonadal hormones can be addressed in studies in which these are administered to neonates. Alternatively, treatment with gonadal hormone receptor agonists or antagonists, and the use of gonadal hormone receptor knockout mice can not only help us to identify which hormones play a role in the sex-bias that is observed but can also lead to the identification of the specific molecular mechanisms involved. The “Four Core Genotypes” (FCG) mouse model offers the unique opportunity to isolate sex chromosome from gonadal hormone contributions to sex-bias. This mouse strain combines two mutations that allow for gonadal determination to be independent from sex chromosome complements. Specifically, the testes determining gene (Sry) was deleted from the Y chromosome (Y^−^) and a Sry transgene (TgSry) was inserted into autosomal chromosome 3, resulting in four genotypes: gonadal females with XX or XY^−^, and gonadal males with XY^−^ TgSry or with XX TgSry (121, 149). This model has been informative in understanding the sex bias in autoimmune diseases such as SLE and multiple sclerosis (150).

## ASD and the microbiome

The gut microbiome is another environmental factor proposed to exert significant modulating effects on ASD susceptibility. Gastrointestinal (GI) disturbances, including increased intestinal permeability (151, 152) and inflammatory bowel disease (153), represent a common comorbidity in individuals with ASD (154, 155). Furthermore, there is a strong correlation between GI complaints and ASD symptom severity (156, 157). Given the effect of the gut microbiota on behavior [(158–164); reviewed by (165)], brain development [(3); reviewed by (165)] and brain gene expression in mice (158, 162–164, 166, 167), alterations of the gut microbiota may not only lead to an increase in the prevalence of GI disturbances in ASD but also promote ASD susceptibility. Indeed, individuals with ASD can have an altered gut microbiota composition (156, 168–173).

The gut microbiota may act in concert with maternal brain-reactive antibodies, genetic variants and sex-specific factors to modulate ASD susceptibility pre- and post-natally. In the model of maternal immune activation (MIA), specific maternal gut microbiota are necessary for the development of MIA-associated behavioral phenotypes and neurodevelopmental abnormalities. Segmented filamentous bacteria (SFB) -specific T_H_17 cells are stimulated by dendritic cells primed by poly (I:C) to produce high levels of IL-17a (174), a key cytokine for ASD-like phenotype induction in the MIA model (175, 176). Furthermore, during fetal development, metabolites produced by the maternal gut microbiota can alter BBB tight junction protein expression thus modifying BBB permeability. Offspring of germ-free mice have a decreased expression of occludin and claudin-5 which contributes to the increased BBB permeability observed from the fetal stage until adulthood (3). This increased BBB permeability makes the offspring more susceptible to the neurological effects of immune molecules including antibodies as these are then more likely to access the brain parenchyma (3). Finally, shifts in offspring microbiota composition can be induced by factors in the *in utero* environment and contribute to the development of ASD-like behavioral deficits. For example, the offspring of poly (I:C) treated mice have an altered gut microbiota diversity which leads to altered serum metabolites and increased IL-6 and gut permeability (177). Hsiao et al. (177) propose that the increased IL-6 expression alters tight junction protein expression, leading to increased gut permeability, and leaking of harmful metabolites into systemic circulation. Post-natal treatment of the offspring with *Bacteroides fragilis* reversed some of the ASD-like behavioral phenotypes and improved gut barrier permeability, possibly by restoring IL-6 expression, which in turn leads to a partial correction of tight junction protein expression (177).

## Conclusions

**Figure 1:**
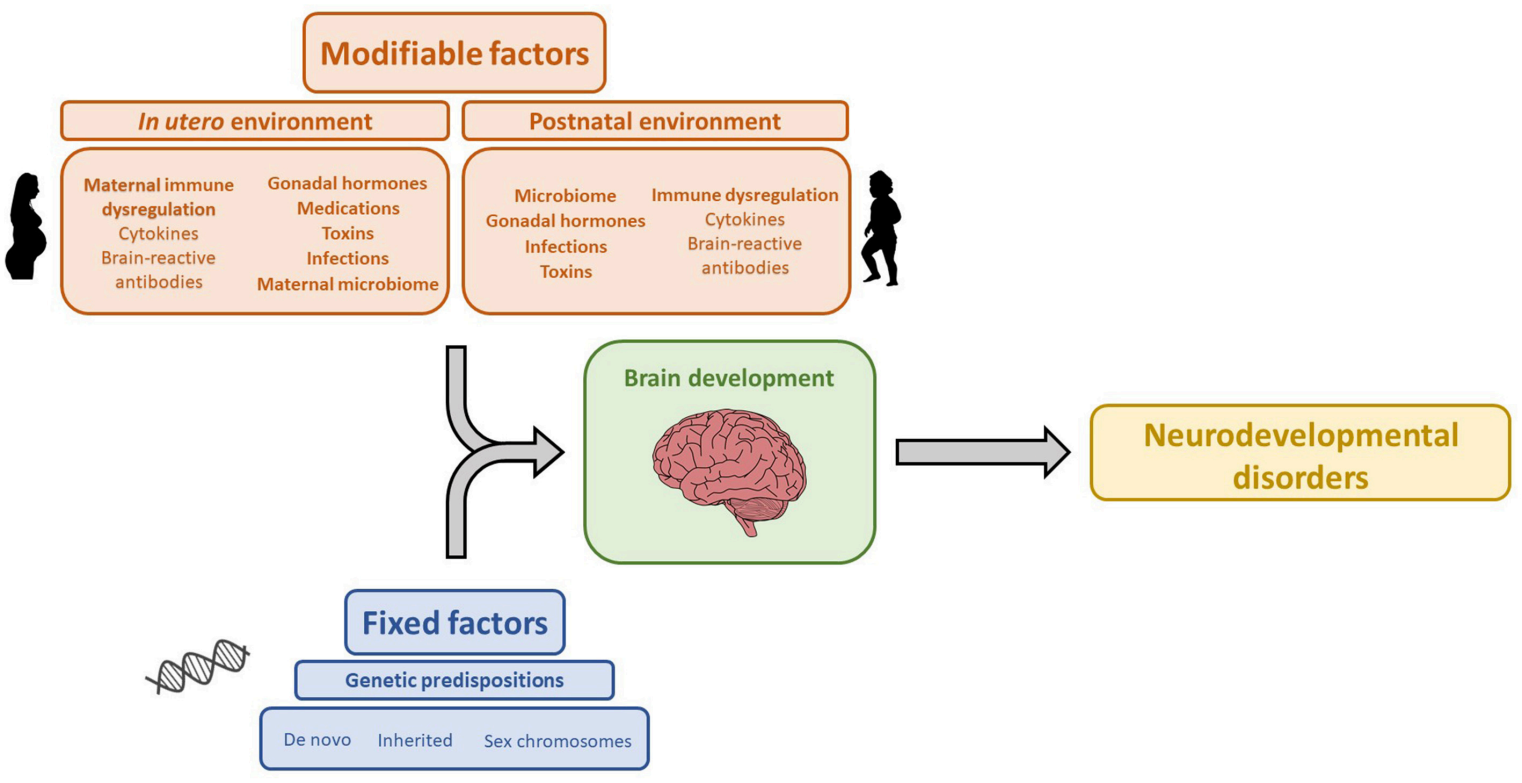
Genetic and environmental factors that influence brain development and contribute to the etiology of neurodevelopmental disorders. The icon images included in this Figure were obtained from openclipart.org, and are images in the Public Domain.

The association between maternal brain-reactive antibodies and the pathogenesis of neurodevelopmental disorders has been well-established by both epidemiologic and animal studies. Maternal autoimmune disease and brain-reactive antibodies have been shown to increase the risk of neurodevelopmental disorders. Moreover, animal studies have shown that *in utero* exposure to maternal brain-reactive antibodies is sufficient to permanently alter brain anatomy and cause aberrant cognition or behavior mimicking certain neurodevelopmental syndromes. Specific neurodevelopmental disorders and the severity of symptomatology are likely determined by an interplay between genetics and environmental risk factors including maternal brain-reactive antibody, maternal cytokines, gonadal hormones, and the microbiome (Figure 1). As the prevalence of neurodevelopmental disorders has been significantly increasing (178), research on the *in utero* environment, including maternal brain-reactive antibodies, is of great biomedical importance. Identifying potentially pathogenic antibodies and understanding their mechanisms of fetal brain injury provide an opportunity to detect and protect fetuses at risk.

## Author Contributions

AG-G contributed to the concept and writing of the manuscript. BD contributed to the concept, writing, and reviewing of the manuscript.

### Conflict of Interest Statement

The authors declare that the research was conducted in the absence of any commercial or financial relationships that could be construed as a potential conflict of interest.

## Abbreviations

ASD: Autism Spectrum Disorder
BBB: Blood Brain Barrier
CNS: Central Nervous System
AD: Autoimmune disease
SLE: Systemic Lupus Erythematosus
NMO: Neuromyelitis Optica
LPS: lipopolysaccharide
NSPA: neuronal surface P antigen
NMDAR: N-methyl-D-aspartate receptors
DNRAb: SLE anti-DNA antibodies that cross-react with N-methyl-D-aspartate receptors
FcRn: neonatal Fc receptor
ADHD: Attention Deficit and Hyperactivity Disorders
DSM-V: Diagnostic and Statistical Manual of Mental Disorders Fifth Edition
ND: neurodevelopmental and neuropsychiatric disorders
NT: neurotypically developing
PPD: Pervasive Developmental Disorders
LD: Learning disabilities
TS: Tourette Syndrome
RA: rheumatoid arthritis
TPOAbs: Thyroid peroxidase antibodies
Abs: antibodies
P: post-natal day
IHC: Immunohistochemistry
NB: Nueroblastoma
WB: Western blot
LDH-A: lactate dehydrogenase A
LDH-B, lactate dehydrogenase B;YBX1: Y-box bonding protein 1
STIP1: stress-induced phosphoprotein 1
CRMP1: collapsing response mediator protein 1
CRMP2: collapsing response mediator protein 2
GDA: guanine deaminase
MSEL: Mullen Scales of Early Learning
VABS: Vineland Adaptive Behavioral Scales
ABC: Aberrant Behavioral Checklist
SVZ: subventricular zone
USVs: ultrasonic vocalizations
CNTNAP2: Contactin Associated Protein-Like 2
HEK: Human embryonic kidney cells
tGFP: turbo Green fluorescent protein
KO: Knockout
CDFE: cortical dysplasia-focal epilepsy
E: embryonic day
IV: intravenous
IP: intraperitoneal
SGZ: subgranular zone
VZ: ventricular zone
FCG: Four Core Genotype
Sry: testes determining gene
Y^−^: Y chromosome with deleted Sry
TgSry: Sry transgene
GI: gastrointestinal
MIA: Maternal Immune Activation
SFB: Segmented filamentous bacteria.

## References

[1] Goldstein GW. Endothelial cell-astrocyte interactions. A cellular model of the blood-brain barrier. Ann N Y Acad Sci. (1988) 529:31–9. 10.1111/j.1749-6632.1988.tb51417.x 3293508

[2] Kowal C Athanassiou A Chen H Diamond B. Maternal antibodies and developing blood-brain barrier. Immunol Res. (2015) 63:18–25. 10.1007/s12026-015-8714-5 26507553 PMC4745584

[3] Braniste V Al-Asmakh M Kowal C Anuar F Abbaspour A Tóth M. The gut microbiota influences blood-brain barrier permeability in mice. Sci Transl Med. (2014) 6:263ra158. 10.1126/scitranslmed.3009759 25411471 PMC4396848

[4] Huerta PT Kowal C DeGiorgio LA Volpe BT Diamond B. Immunity and behavior: antibodies alter emotion. Proc Natl Acad Sci USA. (2006) 103:678–83. 10.1073/pnas.0510055103 16407105 PMC1334673

[5] Kowal C DeGiorgio LA Nakaoka T Hetherington H Huerta PT Diamond B. Cognition and immunity; antibody impairs memory. Immunity. (2004) 21:179–88. 10.1016/j.immuni.2004.07.011 15308099

[6] Tsao N Hsu HP Wu CM Liu CC Lei HY. Tumour necrosis factor-alpha causes an increase in blood-brain barrier permeability during sepsis. J Med Microbiol. (2001) 50:812–21. 10.1099/0022-1317-50-9-812 11549183

[7] Ballabh P Braun A Nedergaard M. The blood-brain barrier: an overview: structure, regulation, and clinical implications. Neurobiol Dis. (2004) 16:1–13. 10.1016/j.nbd.2003.12.016 15207256

[8] Matsueda Y Arinuma Y Nagai T Hirohata S. Elevation of serum anti-glucose-regulated protein 78 antibodies in neuropsychiatric systemic lupus erythematosus. Lupus Sci Med. (2018) 5:e000281. 10.1136/lupus-2018-000281 30397496 PMC6203046

[9] Montagne A Barnes SR Sweeney MD Halliday MR Sagare AP Zhao Z. Blood-brain barrier breakdown in the aging human hippocampus. Neuron. (2015) 85:296–302. 10.1016/j.neuron.2014.12.032 25611508 PMC4350773

[10] Thom G Hatcher J Hearn A Paterson J Rodrigo N Beljean A. Isolation of blood-brain barrier-crossing antibodies from a phage display library by competitive elution and their ability to penetrate the central nervous system. MAbs. (2018) 10:304–14. 10.1080/19420862.2017.1409320 29182455 PMC5825204

[11] Wylezinski LS Hawiger J. Interleukin 2 activates brain microvascular endothelial cells resulting in destabilization of adherens junctions. J Biol Chem. (2016) 291:22913–23. 10.1074/jbc.M116.729038 27601468 PMC5087713

[12] Yoshio T Okamoto H Hirohata S Minota S. IgG anti-NR2 glutamate receptor autoantibodies from patients with systemic lupus erythematosus activate endothelial cells. Arthritis Rheum. (2013) 65:457–63. 10.1002/art.37745 23055186

[13] Diamond B Honig G Mader S Brimberg L Volpe BT. Brain-reactive antibodies and disease. Annu Rev Immunol. (2013) 31:345–85. 10.1146/annurev-immunol-020711-075041 23516983 PMC4401150

[14] Arinuma Y Yanagida T Hirohata S. Association of cerebrospinal fluid anti-NR2 glutamate receptor antibodies with diffuse neuropsychiatric systemic lupus erythematosus. Arthritis Rheum. (2008) 58:1130–5. 10.1002/art.23399 18383393

[15] Bravo-Zehnder M Toledo EM Segovia-Miranda F Serrano FG Benito MJ Metz C. Anti-ribosomal P protein autoantibodies from patients with neuropsychiatric lupus impair memory in mice. Arthritis Rheumatol. (2015) 67:204–14. 10.1002/art.38900 25302407

[16] DeGiorgio LA Konstantinov KN Lee SC Hardin JA Volpe BT Diamond B. A subset of lupus anti-DNA antibodies cross-reacts with the NR2 glutamate receptor in systemic lupus erythematosus. Nat Med. (2001) 7:1189–93. 10.1038/nm1101-1189 11689882

[17] Faust TW Chang EH Kowal C Berlin R Gazaryan IG Bertini E. Neurotoxic lupus autoantibodies alter brain function through two distinct mechanisms. Proc Natl Acad Sci USA. (2010) 107:18569–74. 10.1073/pnas.1006980107 20921396 PMC2972998

[18] Harrison MJ Ravdin LD Lockshin MD. Relationship between serum NR2a antibodies and cognitive dysfunction in systemic lupus erythematosus. Arthritis Rheum. (2006) 54:2515–22. 10.1002/art.22030 16868972

[19] Matus S Burgos PV Bravo-Zehnder M Kraft R Porras OH Farías P. Antiribosomal-P autoantibodies from psychiatric lupus target a novel neuronal surface protein causing calcium influx and apoptosis. J Exp Med. (2007) 204:3221–34. 10.1084/jem.20071285 18056288 PMC2150977

[20] Omdal R Brokstad K Waterloo K Koldingsnes W Jonsson R Mellgren SI. Neuropsychiatric disturbances in SLE are associated with antibodies against NMDA receptors. Eur J Neurol. (2005) 12:392–8. 10.1111/j.1468-1331.2004.00976.x 15804272

[21] Alaedini A Okamoto H Briani C Wollenberg K Shill HA Bushara KO. Immune cross-reactivity in celiac disease: anti-gliadin antibodies bind to neuronal synapsin I. J Immunol. (2007) 178:6590–5. 10.4049/jimmunol.178.10.6590 17475890

[22] Hadjivassiliou M Aeschlimann P Strigun A Sanders DS Woodroofe N Aeschlimann D. Autoantibodies in gluten ataxia recognize a novel neuronal transglutaminase. Ann Neurol. (2008) 64:332–43. 10.1002/ana.21450 18825674

[23] Lennon VA Kryzer TJ Pittock SJ Verkman AS Hinson SR. IgG marker of optic-spinal multiple sclerosis binds to the aquaporin-4 water channel. J Exp Med. (2005) 202:473–7. 10.1084/jem.20050304 16087714 PMC2212860

[24] Mader S Lutterotti A Di Pauli F Kuenz B Schanda K Aboul-Enein F. Patterns of antibody binding to aquaporin-4 isoforms in neuromyelitis optica. PLoS ONE. (2010) 5:e10455. 10.1371/journal.pone.0010455 20463974 PMC2864757

[25] Hinson SR McKeon A Fryer JP Apiwattanakul M Lennon VA Pittock SJ. Prediction of neuromyelitis optica attack severity by quantitation of complement-mediated injury to aquaporin-4-expressing cells. Arch Neurol. (2009) 66:1164–7. 10.1001/archneurol.2009.188 19752309

[26] Zhang H Bennett JL Verkman AS. *Ex vivo* spinal cord slice model of neuromyelitis optica reveals novel immunopathogenic mechanisms. Ann Neurol. (2011) 70:943–54. 10.1002/ana.22551 22069219 PMC3319401

[27] Darnell RB Posner JB. Paraneoplastic syndromes involving the nervous system. N Engl J Med. (2003) 349:1543–54. 10.1056/NEJMra023009 14561798

[28] Gultekin SH Rosenfeld MR Voltz R Eichen J Posner JB Dalmau J. Paraneoplastic limbic encephalitis: neurological symptoms, immunological findings and tumour association in 50 patients. Brain. (2000) 123 (Pt 7):1481–94. 10.1093/brain/123.7.1481 10869059

[29] Rojas-Marcos I Rousseau A Keime-Guibert F Reñé R Cartalat-Carel S Delattre JY. Spectrum of paraneoplastic neurologic disorders in women with breast and gynecologic cancer. Medicine. (2003) 82:216–23. 10.1097/01.md.0000076004.64510.ce 12792307

[30] Bataller L Wade DF Graus F Stacey HD Rosenfeld MR Dalmau J. Antibodies to Zic4 in paraneoplastic neurologic disorders and small-cell lung cancer. Neurology. (2004) 62:778–82. 10.1212/01.WNL.0000113749.77217.01 15007130 PMC2574539

[31] Dalmau J Tüzün E Wu HY Masjuan J Rossi JE Voloschin A. Paraneoplastic anti-N-methyl-D-aspartate receptor encephalitis associated with ovarian teratoma. Ann Neurol. (2007) 61:25–36. 10.1002/ana.21050 17262855 PMC2430743

[32] Dalmau J Lancaster E Martinez-Hernandez E Rosenfeld MR Balice-Gordon R. Clinical experience and laboratory investigations in patients with anti-NMDAR encephalitis. Lancet Neurol. (2011) 10:63–74. 10.1016/S1474-4422(10)70253-2 21163445 PMC3158385

[33] Butler MH Hayashi A Ohkoshi N Villmann C Becker CM Feng G. Autoimmunity to gephyrin in Stiff-Man syndrome. Neuron. (2000) 26:307–12. 10.1016/S0896-6273(00)81165-4 10839351

[34] Hernández-Echebarría L Saiz A Arés A Tejada J García-Tuñón L Nieves C. Paraneoplastic encephalomyelitis associated with pancreatic tumor and anti-GAD antibodies. Neurology. (2006) 66:450–1. 10.1212/01.wnl.0000196488.87746.7b 16476955

[35] Saiz A Blanco Y Sabater L González F Bataller L Casamitjana R. Spectrum of neurological syndromes associated with glutamic acid decarboxylase antibodies: diagnostic clues for this association. Brain. (2008) 131(Pt 10):2553–63. 10.1093/brain/awn183 18687732

[36] Shams'ili S Grefkens J de Leeuw B van den Bent M Hooijkaas H van der Holt B. Paraneoplastic cerebellar degeneration associated with antineuronal antibodies: analysis of 50 patients. Brain. (2003) 126(Pt 6):1409–18. 10.1093/brain/awg133 12764061

[37] Jernigan M Morcos Y Lee SM Dohan FC Raine C Levin MC. IgG in brain correlates with clinicopathological damage in HTLV-1 associated neurologic disease. Neurology. (2003) 60:1320–7. 10.1212/01.WNL.0000059866.03880.BA 12707436

[38] Kalume F Lee SM Morcos Y Callaway JC Levin MC. Molecular mimicry: cross-reactive antibodies from patients with immune-mediated neurologic disease inhibit neuronal firing. J Neurosci Res. (2004) 77:82–9. 10.1002/jnr.20137 15197740

[39] Amevigbe MD Jauberteau-Marchan MO Bouteille B Doua F Breton JC Nicolas JA. Human African trypanosomiasis: presence of antibodies to galactocerebrosides. Am J Trop Med Hyg. (1992) 47:652–62. 10.4269/ajtmh.1992.47.652 1449206

[40] Asonganyi T Lando G Ngu JL. Serum antibodies against human brain myelin proteins in Gambian trypanosomiasis. Ann Soc Belg Med Trop. (1989) 69:213–21. 2692525

[41] Hunter CA Jennings FW Tierney JF Murray M Kennedy PG. Correlation of autoantibody titres with central nervous system pathology in experimental African trypanosomiasis. J Neuroimmunol. (1992) 41:143–8. 10.1016/0165-5728(92)90064-R 1281824

[42] Brimberg L Benhar I Mascaro-Blanco A Alvarez K Lotan D Winter C. Behavioral, pharmacological, and immunological abnormalities after streptococcal exposure: a novel rat model of Sydenham chorea and related neuropsychiatric disorders. Neuropsychopharmacology. (2012) 37:2076–87. 10.1038/npp.2012.56 22534626 PMC3398718

[43] Garvey MA Snider LA Leitman SF Werden R Swedo SE Treatment of Sydenham's chorea with intravenous immunoglobulin, plasma exchange, or prednisone. J Child Neurol. (2005) 20:424–9. 10.1177/08830738050200050601 15968928

[44] Kirvan CA Swedo SE Heuser JS Cunningham MW. Mimicry and autoantibody-mediated neuronal cell signaling in Sydenham chorea. Nat Med. (2003) 9:914–20. 10.1038/nm892 12819778

[45] Kirvan CA Swedo SE Kurahara D Cunningham MW. Streptococcal mimicry and antibody-mediated cell signaling in the pathogenesis of Sydenham's chorea. Autoimmunity. (2006) 39:21–9. 10.1080/08916930500484757 16455579

[46] Kirvan CA Cox CJ Swedo SE Cunningham MW. Tubulin is a neuronal target of autoantibodies in Sydenham's chorea. J Immunol. (2007) 178:7412–21. 10.4049/jimmunol.178.11.7412 17513792

[47] Yaddanapudi K Hornig M Serge R De Miranda J Baghban A Villar G. Passive transfer of streptococcus-induced antibodies reproduces behavioral disturbances in a mouse model of pediatric autoimmune neuropsychiatric disorders associated with streptococcal infection. Mol Psychiatry. (2010) 15:712–26. 10.1038/mp.2009.77 19668249

[48] Dewing P Shi T Horvath S Vilain E. Sexually dimorphic gene expression in mouse brain precedes gonadal differentiation. Brain Res Mol Brain Res. (2003) 118:82–90. 10.1016/S0169-328X(03)00339-5 14559357

[49] Trabzuni D Ramasamy A Imran S Walker R Smith C Weale ME. Widespread sex differences in gene expression and splicing in the adult human brain. Nat Commun. (2013) 4:2771. 10.1038/ncomms3771 24264146 PMC3868224

[50] Xu J Burgoyne PS Arnold AP. Sex differences in sex chromosome gene expression in mouse brain. Hum Mol Genet. (2002) 11:1409–19. 10.1093/hmg/11.12.1409 12023983

[51] Katzav A Ben-Ziv T Chapman J Blank M Reichlin M Shoenfeld Y. Anti-P ribosomal antibodies induce defect in smell capability in a model of CNS -SLE (depression). J Autoimmun. (2008) 31:393–8. 10.1016/j.jaut.2008.09.002 18947972

[52] Katzav A Solodeev I Brodsky O Chapman J Pick CG Blank M. Induction of autoimmune depression in mice by anti-ribosomal P antibodies via the limbic system. Arthritis Rheum. (2007) 56:938–48. 10.1002/art.22419 17328071

[53] Gluecksohn-Waelsch S The effect of maternal immunization against organ tissues on embryonic differentiation in the mouse. J Embryol Exp Morphol. (1957) 5:83.

[54] Karpiak SE Rapport MM. Behavioral changes in 2-month-old rats following prenatal exposure to antibodies against synaptic membranes. Brain Res. (1975) 92:405–13. 10.1016/0006-8993(75)90325-X 1174960

[55] McAllister DL Kaplan BJ Edworthy SM Martin L Crawford SG Ramsey-Goldman R. The influence of systemic lupus erythematosus on fetal development: cognitive, behavioral, and health trends. J Int Neuropsychol Soc. (1997) 3:370–6. 9260446

[56] Ross G Sammaritano L Nass R Lockshin M. Effects of mothers' autoimmune disease during pregnancy on learning disabilities and hand preference in their children. Arch Pediatr Adolesc Med. (2003) 157:397–402. 10.1001/archpedi.157.4.397 12695238

[57] Urowitz MB Gladman DD MacKinnon A Ibañez D Bruto V Rovet J. Neurocognitive abnormalities in offspring of mothers with systemic lupus erythematosus. Lupus. (2008) 17:555–60. 10.1177/0961203308089326 18539709

[58] Dalsgaard S Waltoft BL Leckman JF Mortensen PB. Maternal history of autoimmune disease and later development of tourette syndrome in offspring. J Am Acad Child Adolesc Psychiatry. (2015) 54:495–501.e491. 10.1016/j.jaac.2015.03.008 26004665

[59] Sweeten TL Bowyer SL Posey DJ Halberstadt GM McDougle CJ. Increased prevalence of familial autoimmunity in probands with pervasive developmental disorders. Pediatrics. (2003) 112:e420. 10.1542/peds.112.5.e420 14595086

[60] Ghassabian A Bongers-Schokking JJ de Rijke YB van Mil N Jaddoe VW de Muinck Keizer-Schrama SM. Maternal thyroid autoimmunity during pregnancy and the risk of attention deficit/hyperactivity problems in children: the Generation R Study. Thyroid. (2012) 22:178–86. 10.1089/thy.2011.0318 22175242 PMC3271370

[61] Atladóttir HO Pedersen MG Thorsen P Mortensen PB Deleuran B Eaton WW. Association of family history of autoimmune diseases and autism spectrum disorders. Pediatrics. (2009) 124:687–94. 10.1542/peds.2008-2445 19581261

[62] Comi AM Zimmerman AW Frye VH Law PA Peeden JN. Familial clustering of autoimmune disorders and evaluation of medical risk factors in autism. J Child Neurol. (1999) 14:388–94. 10.1177/088307389901400608 10385847

[63] Croen LA Grether JK Yoshida CK Odouli R Van de Water J. Maternal autoimmune diseases, asthma and allergies, and childhood autism spectrum disorders: a case-control study. Arch Pediatr Adolesc Med. (2005) 159:151–7. 10.1001/archpedi.159.2.151 15699309

[64] Mostafa GA Shehab AA. The link of C4B null allele to autism and to a family history of autoimmunity in Egyptian autistic children. J Neuroimmunol. (2010) 223:115–9. 10.1016/j.jneuroim.2010.03.025 20452682

[65] Mostafa GA El-Sherif DF Al-Ayadhi LY. Systemic auto-antibodies in children with autism. J Neuroimmunol. (2014) 272:94–8. 10.1016/j.jneuroim.2014.04.011 24837704

[66] Vinet É Pineau CA Clarke AE Scott S Fombonne É Joseph L. Increased risk of autism spectrum disorders in children born to women with systemic lupus erythematosus: results from a large population-based cohort. Arthritis Rheumatol. (2015) 67:3201–8. 10.1002/art.39320 26315754

[67] Brimberg L Sadiq A Gregersen PK Diamond B. Brain-reactive IgG correlates with autoimmunity in mothers of a child with an autism spectrum disorder. Mol Psychiatry. (2013) 18:1171–7. 10.1038/mp.2013.101 23958959

[68] Kim J Mohanty S Ganesan LP Hua K Jarjoura D Hayton WL. FcRn in the yolk sac endoderm of mouse is required for IgG transport to fetus. J Immunol. (2009) 182:2583–9. 10.4049/jimmunol.0803247 19234152 PMC2676880

[69] Malek A Sager R Kuhn P Nicolaides KH Schneider H. Evolution of maternofetal transport of immunoglobulins during human pregnancy. Am J Reprod Immunol. (1996) 36:248–55. 10.1111/j.1600-0897.1996.tb00172.x 8955500

[70] Palmeira P Quinello C Silveira-Lessa AL Zago CA Carneiro-Sampaio M. IgG placental transfer in healthy and pathological pregnancies. Clin Dev Immunol. (2012) 2012:985646. 10.1155/2012/985646 22235228 PMC3251916

[71] Sarvas H Seppälä I Kurikka S Siegberg R Mäkelä O. Half-life of the maternal IgG1 allotype in infants. J Clin Immunol. (1993) 13:145–51. 10.1007/BF00919271 8320311

[72] Diamond B Huerta PT Mina-Osorio P Kowal C Volpe BT. Losing your nerves? Maybe it's the antibodies. Nat Rev Immunol. (2009) 9:449–56. 10.1038/nri2529 19424277 PMC2783680

[73] American Psychiatric Association and American Psychiatric Association. DSM-5 Task Force (2013). Diagnostic and Statistical Manual of Mental Disorders: DSM-5. 5th ed. Washington, DC: American Psychiatric Association. 10.1176/appi.books.9780890425596

[74] Baio J Wiggins L Christensen DL Maenner MJ Daniels J Warren Z. Prevalence of autism spectrum disorder among children aged 8 years - autism and developmental disabilities monitoring network, 11 sites, United States, 2014. MMWR Surveill Summ. (2018) 67:1–23. 10.15585/mmwr.ss6706a1 29701730 PMC5919599

[75] Bernier R Golzio C Xiong B Stessman HA Coe BP Penn O. Disruptive CHD8 mutations define a subtype of autism early in development. Cell. (2014) 158:263–76. 10.1016/j.cell.2014.06.017 24998929 PMC4136921

[76] Sebat J Lakshmi B Malhotra D Troge J Lese-Martin C Walsh T. Strong association of *de novo* copy number mutations with autism. Science. (2007) 316:445–9. 10.1126/science.1138659 17363630 PMC2993504

[77] Betancur C. Etiological heterogeneity in autism spectrum disorders: more than 100 genetic and genomic disorders and still counting. Brain Res. (2011) 1380:42–77. 10.1016/j.brainres.2010.11.078 21129364

[78] Hallmayer J Cleveland S Torres A Phillips J Cohen B Torigoe T. Genetic heritability and shared environmental factors among twin pairs with autism. Arch Gen Psychiatry. (2011) 68:1095–102. 10.1001/archgenpsychiatry.2011.76 21727249 PMC4440679

[79] Dalton P Deacon R Blamire A Pike M McKinlay I Stein J. Maternal neuronal antibodies associated with autism and a language disorder. Ann Neurol. (2003) 53:533–7. 10.1002/ana.10557 12666123

[80] Croen LA Braunschweig D Haapanen L Yoshida CK Fireman B Grether JK. Maternal mid-pregnancy autoantibodies to fetal brain protein: the early markers for autism study. Biol Psychiatry. (2008) 64:583–8. 10.1016/j.biopsych.2008.05.006 18571628 PMC2574992

[81] Braunschweig D Ashwood P Krakowiak P Hertz-Picciotto I Hansen R Croen LA. Autism: maternally derived antibodies specific for fetal brain proteins. Neurotoxicology. (2008) 29:226–31. 10.1016/j.neuro.2007.10.010 18078998 PMC2305723

[82] Braunschweig D Golub MS Koenig CM Qi L Pessah IN Van de Water J. Maternal autism-associated IgG antibodies delay development and produce anxiety in a mouse gestational transfer model. J Neuroimmunol. (2012) 252:56–65. 10.1016/j.jneuroim.2012.08.002 22951357 PMC4096980

[83] Braunschweig D Krakowiak P Duncanson P Boyce R Hansen RL Ashwood P. Autism-specific maternal autoantibodies recognize critical proteins in developing brain. Transl Psychiatry. (2013) 3:e277. 10.1038/tp.2013.50 23838888 PMC3731784

[84] Edmiston E Jones KL Vu T Ashwood P Van de Water J. Identification of the antigenic epitopes of maternal autoantibodies in autism spectrum disorders. Brain Behav Immun. (2018) 69:399–407. 10.1016/j.bbi.2017.12.014 29289663 PMC5857423

[85] Piras IS Haapanen L Napolioni V Sacco R Van de Water J Persico AM. Anti-brain antibodies are associated with more severe cognitive and behavioral profiles in Italian children with Autism Spectrum Disorder. Brain Behav Immun. (2014) 38:91–9. 10.1016/j.bbi.2013.12.020 24389156 PMC4111628

[86] Rossi CC Fuentes J Van de Water J Amaral DG Brief report: antibodies reacting to brain tissue in basque spanish children with autism spectrum disorder and their mothers. J Autism Dev Disord. (2014) 44:459–465. 10.1007/s10803-013-1859-y PMC3980136 24022729

[87] Nordahl CW Braunschweig D Iosif AM Lee A Rogers S Ashwood P. Maternal autoantibodies are associated with abnormal brain enlargement in a subgroup of children with autism spectrum disorder. Brain Behav Immun. (2013) 30:61–5. 10.1016/j.bbi.2013.01.084 23395715 PMC3641177

[88] Singer HS Morris CM Gause CD Gillin PK Crawford S Zimmerman AW. Antibodies against fetal brain in sera of mothers with autistic children. J Neuroimmunol. (2008) 194:165–72. 10.1016/j.jneuroim.2007.11.004 18093664

[89] Zimmerman AW Connors SL Matteson KJ Lee LC Singer HS Castaneda JA. Maternal antibrain antibodies in autism. Brain Behav Immun. (2007) 21:351–7. 10.1016/j.bbi.2006.08.005 17029701

[90] Ali NH Khalaf SK Al-Asadi JN Abed AH. Maternal antineuronal antibodies and risk of childhood autism spectrum disorders: a case-control study. J Chin Med Assoc. (2016) 79:661–4. 10.1016/j.jcma.2016.08.003 27686499

[91] Brimberg L Mader S Jeganathan V Berlin R Coleman TR Gregersen PK. Caspr2-reactive antibody cloned from a mother of an ASD child mediates an ASD-like phenotype in mice. Mol Psychiatry. (2016) 21:1663–71. 10.1038/mp.2016.165 27698429 PMC5583730

[92] Martin LA Ashwood P Braunschweig D Cabanlit M Van de Water J Amaral DG. Stereotypies and hyperactivity in rhesus monkeys exposed to IgG from mothers of children with autism. Brain Behav Immun. (2008) 22:806–16. 10.1016/j.bbi.2007.12.007 18262386 PMC3779644

[93] Braunschweig D Duncanson P Boyce R Hansen R Ashwood P Pessah IN. Behavioral correlates of maternal antibody status among children with autism. J Autism Dev Disord. (2012) 42:1435–45. 10.1007/s10803-011-1378-7 22012245 PMC4871696

[94] Bauman MD Iosif AM Ashwood P Braunschweig D Lee A Schumann CM. Maternal antibodies from mothers of children with autism alter brain growth and social behavior development in the rhesus monkey. Transl Psychiatry. (2013) 3:e278. 10.1038/tp.2013.47 23838889 PMC3731783

[95] Camacho J Jones K Miller E Ariza J Noctor S Van de Water J. Embryonic intraventricular exposure to autism-specific maternal autoantibodies produces alterations in autistic-like stereotypical behaviors in offspring mice. Behav Brain Res. (2014) 266:46–51. 10.1016/j.bbr.2014.02.045 24613242 PMC4075424

[96] Martínez-Cerdeño V Camacho J Fox E Miller E Ariza J Kienzle D. Prenatal exposure to autism-specific maternal autoantibodies alters proliferation of cortical neural precursor cells, enlarges brain, and increases neuronal size in adult animals. Cereb Cortex. (2016) 26:374–83. 10.1093/cercor/bhu291 25535268 PMC4677982

[97] Ariza J Hurtado J Rogers H Ikeda R Dill M Steward C. Maternal autoimmune antibodies alter the dendritic arbor and spine numbers in the infragranular layers of the cortex. PLoS ONE. (2017) 12:e0183443. 10.1371/journal.pone.0183443 28820892 PMC5562324

[98] Jones KL Pride MC Edmiston E Yang M Silverman JL Crawley JN. Autism-specific maternal autoantibodies produce behavioral abnormalities in an endogenous antigen-driven mouse model of autism. Mol Psychiatry. (2018). 10.1038/s41380-018-0126-1. [Epub ahead of print] 29955164 PMC6310680

[99] Singer HS Morris C Gause C Pollard M Zimmerman AW Pletnikov M. Prenatal exposure to antibodies from mothers of children with autism produces neurobehavioral alterations: a pregnant dam mouse model. J Neuroimmunol. (2009) 211:39–48. 10.1016/j.jneuroim.2009.03.011 19362378

[100] Kadam SD French BM Kim ST Morris-Berry CM Zimmerman AW Blue ME. Altered postnatal cell proliferation in brains of mouse pups prenatally exposed to IgG from mothers of children with autistic disorder. J Exp Neurosci. (2013) 7:93–9. 10.4137/JEN.S12979 25157212 PMC4089726

[101] Lee JY Huerta PT Zhang J Kowal C Bertini E Volpe BT. Neurotoxic autoantibodies mediate congenital cortical impairment of offspring in maternal lupus. Nat Med. (2009) 15:91–6. 10.1038/nm.1892 19079257 PMC2615794

[102] Wang L Zhou D Lee J Niu H Faust TW Frattini S. Female mouse fetal loss mediated by maternal autoantibody. J Exp Med. (2012) 209:1083–9. 10.1084/jem.20111986 22565825 PMC3371726

[103] Bel C Oguievetskaia K Pitaval C Goutebroze L Faivre-Sarrailh C. Axonal targeting of Caspr2 in hippocampal neurons via selective somatodendritic endocytosis. J Cell Sci. (2009) 122(Pt 18):3403–13. 10.1242/jcs.050526 19706678

[104] Poliak S Gollan L Martinez R Custer A Einheber S Salzer JL. Caspr2, a new member of the neurexin superfamily, is localized at the juxtaparanodes of myelinated axons and associates with K+ channels. Neuron. (1999) 24:1037–47. 10.1016/S0896-6273(00)81049-1 10624965

[105] Alarcón M Abrahams BS Stone JL Duvall JA Perederiy JV Bomar JM. Linkage, association, and gene-expression analyses identify CNTNAP2 as an autism-susceptibility gene. Am J Hum Genet. (2008) 82:150–9. 10.1016/j.ajhg.2007.09.005 18179893 PMC2253955

[106] Arking DE Cutler DJ Brune CW Teslovich TM West K Ikeda M. A common genetic variant in the neurexin superfamily member CNTNAP2 increases familial risk of autism. Am J Hum Genet. (2008) 82:160–4. 10.1016/j.ajhg.2007.09.015 18179894 PMC2253968

[107] Bakkaloglu B O'Roak BJ Louvi A Gupta AR Abelson JF Morgan TM. Molecular cytogenetic analysis and resequencing of contactin associated protein-like 2 in autism spectrum disorders. Am J Hum Genet. (2008) 82:165–73. 10.1016/j.ajhg.2007.09.017 18179895 PMC2253974

[108] Newbury DF Paracchini S Scerri TS Winchester L Addis L Richardson AJ. Investigation of dyslexia and SLI risk variants in reading- and language-impaired subjects. Behav Genet. (2011) 41:90–104. 10.1007/s10519-010-9424-3 21165691 PMC3029677

[109] Nord AS Roeb W Dickel DE Walsh T Kusenda M O'Connor KL. Reduced transcript expression of genes affected by inherited and *de novo* CNVs in autism. Eur J Hum Genet. (2011) 19:727–31. 10.1038/ejhg.2011.24 21448237 PMC3110052

[110] O'Roak BJ Deriziotis P Lee C Vives L Schwartz JJ Girirajan S. Exome sequencing in sporadic autism spectrum disorders identifies severe *de novo* mutations. Nat Genet. (2011) 43:585–9. 10.1038/ng.835 21572417 PMC3115696

[111] Poot M Beyer V Schwaab I Damatova N Van't Slot R Prothero J. Disruption of CNTNAP2 and additional structural genome changes in a boy with speech delay and autism spectrum disorder. Neurogenetics. (2010) 11:81–9. 10.1007/s10048-009-0205-1 19582487

[112] Sehested LT Møller RS Bache I Andersen NB Ullmann R Tommerup N. Deletion of 7q34-q36.2 in two siblings with mental retardation, language delay, primary amenorrhea, and dysmorphic features. Am J Med Genet A. (2010) 152A:3115–9. 10.1002/ajmg.a.33476 21082657

[113] Steer CD Golding J Bolton PF. Traits contributing to the autistic spectrum. PLoS ONE. (2010) 5:e12633. 10.1371/journal.pone.0012633 20838614 PMC2935882

[114] Strauss KA Puffenberger EG Huentelman MJ Gottlieb S Dobrin SE Parod JM. Recessive symptomatic focal epilepsy and mutant contactin-associated protein-like 2. N Engl J Med. (2006) 354:1370–7. 10.1056/NEJMoa052773 16571880

[115] Vernes SC Newbury DF Abrahams BS Winchester L Nicod J Groszer M. A functional genetic link between distinct developmental language disorders. N Engl J Med. (2008) 359:2337–45. 10.1056/NEJMoa0802828 18987363 PMC2756409

[116] Peñagarikano O Abrahams BS Herman EI Winden KD Gdalyahu A Dong H. Absence of CNTNAP2 leads to epilepsy, neuronal migration abnormalities, and core autism-related deficits. Cell. (2011) 147:235–46. 10.1016/j.cell.2011.08.040 21962519 PMC3390029

[117] Baron-Cohen S Lombardo MV Auyeung B Ashwin E Chakrabarti B Knickmeyer R. Why are autism spectrum conditions more prevalent in males? PLoS Biol. (2011) 9:e1001081. 10.1371/journal.pbio.1001081 21695109 PMC3114757

[118] Nolen-Hoeksema S Girgus JS. The emergence of gender differences in depression during adolescence. Psychol Bull. (1994) 115:424–43. 10.1037/0033-2909.115.3.424 8016286

[119] Rutter M Caspi A Moffitt TE. Using sex differences in psychopathology to study causal mechanisms: unifying issues and research strategies. J Child Psychol Psychiatry. (2003) 44:1092–115. 10.1111/1469-7610.00194 14626453

[120] Dewing P Chiang CW Sinchak K Sim H Fernagut PO Kelly S. Direct regulation of adult brain function by the male-specific factor SRY. Curr Biol. (2006) 16:415–20. 10.1016/j.cub.2006.01.017 16488877

[121] De Vries GJ Rissman EF Simerly RB Yang LY Scordalakes EM Auger CJ. A model system for study of sex chromosome effects on sexually dimorphic neural and behavioral traits. J Neurosci. (2002) 22:9005–14. 10.1523/JNEUROSCI.22-20-09005.2002 12388607 PMC6757680

[122] Carruth LL Reisert I Arnold AP. Sex chromosome genes directly affect brain sexual differentiation. Nat Neurosci. (2002) 5:933–4. 10.1038/nn922 12244322

[123] Cox KH Rissman EF. Sex differences in juvenile mouse social behavior are influenced by sex chromosomes and social context. Genes Brain Behav. (2011) 10:465–72. 10.1111/j.1601-183X.2011.00688.x 21414140 PMC3107935

[124] McPhie-Lalmansingh AA Tejada LD Weaver JL Rissman EF. Sex chromosome complement affects social interactions in mice. Horm Behav. (2008) 54:565–70. 10.1016/j.yhbeh.2008.05.016 18590732 PMC2561329

[125] Gatewood JD Wills A Shetty S Xu J Arnold AP Burgoyne PS. Sex chromosome complement and gonadal sex influence aggressive and parental behaviors in mice. J Neurosci. (2006) 26:2335–42. 10.1523/JNEUROSCI.3743-05.2006 16495461 PMC6674813

[126] Maxson SC Didier-Erickson A Ogawa S. The Y chromosome, social signals, and offense in mice. Behav Neural Biol. (1989) 52:251–9. 10.1016/S0163-1047(89)90369-5 2803176

[127] Sluyter F Bohus B Beldhuis HJ van Oortmerssen GA. Autosomal and Y chromosomal effects on the stereotyped response to apomorphine in wild house mice. Pharmacol Biochem Behav. (1995) 52:17–22. 10.1016/0091-3057(95)00092-B 7501661

[128] Kopsida E Lynn PM Humby T Wilkinson LS Davies W. Dissociable effects of Sry and sex chromosome complement on activity, feeding and anxiety-related behaviours in mice. PLoS ONE. (2013) 8:e73699. 10.1371/journal.pone.0073699 24009762 PMC3751882

[129] Carrel L Cottle AA Goglin KC Willard HF. A first-generation X-inactivation profile of the human X chromosome. Proc Natl Acad Sci USA. (1999) 96:14440–4. 10.1073/pnas.96.25.14440 10588724 PMC24455

[130] Carrel L Willard HF. X-inactivation profile reveals extensive variability in X-linked gene expression in females. Nature. (2005) 434:400–4. 10.1038/nature03479 15772666

[131] Yang F Babak T Shendure J Disteche CM. Global survey of escape from X inactivation by RNA-sequencing in mouse. Genome Res. (2010) 20:614–22. 10.1101/gr.103200.109 20363980 PMC2860163

[132] Gregg C Zhang J Butler JE Haig D Dulac C. Sex-specific parent-of-origin allelic expression in the mouse brain. Science. (2010) 329:682–5. 10.1126/science.1190831 20616234 PMC2997643

[133] Raefski AS O'Neill MJ. Identification of a cluster of X-linked imprinted genes in mice. Nat Genet. (2005) 37:620–4. 10.1038/ng1567 15908953

[134] Skuse DH James RS Bishop DV Coppin B Dalton P Aamodt-Leeper G. Evidence from Turner's syndrome of an imprinted X-linked locus affecting cognitive function. Nature. (1997) 387:705–8. 10.1038/42706 9192895

[135] Arai Y Sekine Y Murakami S. Estrogen and apoptosis in the developing sexually dimorphic preoptic area in female rats. Neurosci Res. (1996) 25:403–7. 10.1016/0168-0102(96)01070-X 8866522

[136] Arnold AP Gorski RA. Gonadal steroid induction of structural sex differences in the central nervous system. Annu Rev Neurosci. (1984) 7:413–42. 10.1146/annurev.ne.07.030184.002213 6370082

[137] Brinton RD Tran J Proffitt P Montoya M. 17 beta-Estradiol enhances the outgrowth and survival of neocortical neurons in culture. Neurochem Res. (1997) 22:1339–51. 10.1023/A:1022015005508 9355106

[138] Gould E Woolley CS Frankfurt M McEwen BS. Gonadal steroids regulate dendritic spine density in hippocampal pyramidal cells in adulthood. J Neurosci. (1990) 10:1286–91. 10.1523/JNEUROSCI.10-04-01286.1990 2329377 PMC6570209

[139] Nilsen J Mor G Naftolin F. Estrogen-regulated developmental neuronal apoptosis is determined by estrogen receptor subtype and the Fas/Fas ligand system. J Neurobiol. (2000) 43:64–78. 10.1002/(SICI)1097-4695(200004)43:1&lt;64::AID-NEU6&gt;3.0.CO;2-7 10756067

[140] Wang L Andersson S Warner M Gustafsson JA. Estrogen receptor (ER)beta knockout mice reveal a role for ERbeta in migration of cortical neurons in the developing brain. Proc Natl Acad Sci USA. (2003) 100:703–8. 10.1073/pnas.242735799 12515851 PMC141060

[141] Wu MV Manoli DS Fraser EJ Coats JK Tollkuhn J Honda S. Estrogen masculinizes neural pathways and sex-specific behaviors. Cell. (2009) 139:61–72. 10.1016/j.cell.2009.07.036 19804754 PMC2851224

[142] Wu MV Shah NM. Control of masculinization of the brain and behavior. Curr Opin Neurobiol. (2011) 21:116–23. 10.1016/j.conb.2010.09.014 20970320 PMC3046257

[143] Knickmeyer R Baron-Cohen S Raggatt P Taylor K. Foetal testosterone, social relationships, and restricted interests in children. J Child Psychol Psychiatry. (2005) 46:198–210. 10.1111/j.1469-7610.2004.00349.x 15679528

[144] Knickmeyer R Baron-Cohen S Raggatt P Taylor K Hackett G. Fetal testosterone and empathy. Horm Behav. (2006) 49:282–92. 10.1016/j.yhbeh.2005.08.010 16226265

[145] Lutchmaya S Baron-Cohen S Raggatt P Foetal testosterone and eye contact in 12-month-old human infants. Infant Behav Dev. (2002) 25:327–35. 10.1016/S0163-6383(02)00094-2

[146] Hoffman EJ Turner KJ Fernandez JM Cifuentes D Ghosh M Ijaz S. Estrogens suppress a behavioral phenotype in zebrafish mutants of the autism risk gene, CNTNAP2. Neuron. (2016) 89:725–33. 10.1016/j.neuron.2015.12.039 26833134 PMC4766582

[147] Macrì S Biamonte F Romano E Marino R Keller F Laviola G. Perseverative responding and neuroanatomical alterations in adult heterozygous reeler mice are mitigated by neonatal estrogen administration. Psychoneuroendocrinology. (2010) 35:1374–87. 10.1016/j.psyneuen.2010.03.012 20452127

[148] vom Saal FS. Sexual differentiation in litter-bearing mammals: influence of sex of adjacent fetuses *in utero*. J Anim Sci. (1989) 67:1824–40. 10.2527/jas1989.6771824x 2670873

[149] Itoh Y Mackie R Kampf K Domadia S Brown JD O'Neill R. Four core genotypes mouse model: localization of the Sry transgene and bioassay for testicular hormone levels. BMC Res Notes. (2015) 8:69. 10.1186/s13104-015-0986-2 25870930 PMC4354741

[150] Smith-Bouvier DL Divekar AA Sasidhar M Du S Tiwari-Woodruff SK King JK. A role for sex chromosome complement in the female bias in autoimmune disease. J Exp Med. (2008) 205:1099–108. 10.1084/jem.20070850 18443225 PMC2373842

[151] D'Eufemia P Celli M Finocchiaro R Pacifico L Viozzi L Zaccagnini M. Abnormal intestinal permeability in children with autism. Acta Paediatr. (1996) 85:1076–9. 10.1111/j.1651-2227.1996.tb14220.x 8888921

[152] de Magistris L Familiari V Pascotto A Sapone A Frolli A Iardino P. Alterations of the intestinal barrier in patients with autism spectrum disorders and in their first-degree relatives. J Pediatr Gastroenterol Nutr. (2010) 51:418–24. 10.1097/MPG.0b013e3181dcc4a5 20683204

[153] Kohane IS McMurry A Weber G MacFadden D Rappaport L Kunkel L. The co-morbidity burden of children and young adults with autism spectrum disorders. PLoS ONE. (2012) 7:e33224. 10.1371/journal.pone.0033224 22511918 PMC3325235

[154] Buie T Campbell DB Fuchs GJ Furuta GT Levy J Vandewater J. Evaluation, diagnosis, and treatment of gastrointestinal disorders in individuals with ASDs: a consensus report. Pediatrics. (2010) 125(Suppl. 1):S1–18. 10.1542/peds.2009-1878C 20048083

[155] Coury DL Ashwood P Fasano A Fuchs G Geraghty M Kaul A. Gastrointestinal conditions in children with autism spectrum disorder: developing a research agenda. Pediatrics. (2012) 130(Suppl. 2):S160–8. 10.1542/peds.2012-0900N 23118247

[156] Adams JB Johansen LJ Powell LD Quig D Rubin RA. Gastrointestinal flora and gastrointestinal status in children with autism–comparisons to typical children and correlation with autism severity. BMC Gastroenterol. (2011) 11:22. 10.1186/1471-230X-11-22 21410934 PMC3072352

[157] McElhanon BO McCracken C Karpen S Sharp WG. Gastrointestinal symptoms in autism spectrum disorder: a meta-analysis. Pediatrics. (2014) 133:872–83. 10.1542/peds.2013-3995 24777214

[158] Arentsen T Raith H Qian Y Forssberg H Diaz Heijtz R. Host microbiota modulates development of social preference in mice. Microb Ecol Health Dis. (2015) 26:29719. 10.3402/mehd.v26.29719 26679775 PMC4683992

[159] Bercik P Park AJ Sinclair D Khoshdel A Lu J Huang X. The anxiolytic effect of Bifidobacterium longum NCC3001 involves vagal pathways for gut-brain communication. Neurogastroenterol Motil. (2011) 23:1132–9. 10.1111/j.1365-2982.2011.01796.x 21988661 PMC3413724

[160] Bravo JA Forsythe P Chew MV Escaravage E Savignac HM Dinan TG. Ingestion of Lactobacillus strain regulates emotional behavior and central GABA receptor expression in a mouse via the vagus nerve. Proc Natl Acad Sci USA. (2011) 108:16050–5. 10.1073/pnas.1102999108 21876150 PMC3179073

[161] Desbonnet L Clarke G Shanahan F Dinan TG Cryan JF. Microbiota is essential for social development in the mouse. Mol Psychiatry. (2014) 19:146–8. 10.1038/mp.2013.65 23689536 PMC3903109

[162] Desbonnet L Clarke G Traplin A O'Sullivan O Crispie F Moloney RD. Gut microbiota depletion from early adolescence in mice: implications for brain and behaviour. Brain Behav Immun. (2015) 48:165–73. 10.1016/j.bbi.2015.04.004 25866195

[163] Diaz Heijtz R Wang S Anuar F Qian Y Björkholm B Samuelsson A. Normal gut microbiota modulates brain development and behavior. Proc Natl Acad Sci USA. (2011) 108:3047–52. 10.1073/pnas.1010529108 21282636 PMC3041077

[164] Neufeld KM Kang N Bienenstock J Foster JA. Reduced anxiety-like behavior and central neurochemical change in germ-free mice. Neurogastroenterol Motil. (2011) 23:255–64, e119. 10.1111/j.1365-2982.2010.01620.x 21054680

[165] Cryan JF Dinan TG. Mind-altering microorganisms: the impact of the gut microbiota on brain and behaviour. Nat Rev Neurosci. (2012) 13:701–12. 10.1038/nrn3346 22968153

[166] Bercik P Denou E Collins J Jackson W Lu J Jury J. The intestinal microbiota affect central levels of brain-derived neurotropic factor and behavior in mice. Gastroenterology. (2011) 141:599–609, 609.e591–3. 10.1053/j.gastro.2011.04.052 21683077

[167] Clarke G Grenham S Scully P Fitzgerald P Moloney RD Shanahan F. The microbiome-gut-brain axis during early life regulates the hippocampal serotonergic system in a sex-dependent manner. Mol Psychiatry. (2013) 18:666–73. 10.1038/mp.2012.77 22688187

[168] Finegold SM Dowd SE Gontcharova V Liu C Henley KE Wolcott RD. Pyrosequencing study of fecal microflora of autistic and control children. Anaerobe. (2010) 16:444–53. 10.1016/j.anaerobe.2010.06.008 20603222

[169] Finegold SM Downes J Summanen PH. Microbiology of regressive autism. Anaerobe. (2012) 18:260–2. 10.1016/j.anaerobe.2011.12.018 22202440

[170] Kang DW Park JG Ilhan ZE Wallstrom G Labaer J Adams JB. Reduced incidence of Prevotella and other fermenters in intestinal microflora of autistic children. PLoS ONE. (2013) 8:e68322. 10.1371/journal.pone.0068322 23844187 PMC3700858

[171] Parracho HM Bingham MO Gibson GR McCartney AL. Differences between the gut microflora of children with autistic spectrum disorders and that of healthy children. J Med Microbiol. (2005) 54(Pt 10):987–91. 10.1099/jmm.0.46101-0 16157555

[172] Williams BL Hornig M Buie T Bauman ML Cho Paik M Wick I. Impaired carbohydrate digestion and transport and mucosal dysbiosis in the intestines of children with autism and gastrointestinal disturbances. PLoS ONE. (2011) 6:e24585. 10.1371/journal.pone.0024585 21949732 PMC3174969

[173] Williams BL Hornig M Parekh T Lipkin WI. Application of novel PCR-based methods for detection, quantitation, and phylogenetic characterization of Sutterella species in intestinal biopsy samples from children with autism and gastrointestinal disturbances. MBio. (2012) 3:e00261-11. 10.1128/mBio.00261-11 22233678 PMC3252763

[174] Kim S Kim H Yim YS Ha S Atarashi K Tan TG. Maternal gut bacteria promote neurodevelopmental abnormalities in mouse offspring. Nature. (2017) 549:528–32. 10.1038/nature23910 28902840 PMC5870873

[175] Choi GB Yim YS Wong H Kim S Kim H Kim SV. The maternal interleukin-17a pathway in mice promotes autism-like phenotypes in offspring. Science. (2016) 351:933–9. 10.1126/science.aad0314 26822608 PMC4782964

[176] Lammert CR Frost EL Bolte AC Paysour MJ Shaw ME Bellinger CE. Cutting edge: critical roles for microbiota-mediated regulation of the immune system in a prenatal immune activation model of autism. J Immunol. (2018) 201:845–50. 10.4049/jimmunol.1701755 29967099 PMC6057827

[177] Hsiao EY McBride SW Hsien S Sharon G Hyde ER McCue T. Microbiota modulate behavioral and physiological abnormalities associated with neurodevelopmental disorders. Cell. (2013) 155:1451–63. 10.1016/j.cell.2013.11.024 24315484 PMC3897394

[178] Boyle CA Boulet S Schieve LA Cohen RA Blumberg SJ Yeargin-Allsopp M. Trends in the prevalence of developmental disabilities in US children, 1997-2008. Pediatrics. (2011) 127:1034–42. 10.1542/peds.2010-2989 21606152

